# Cellulose nanofiber backboned Prussian blue nanoparticles as powerful adsorbents for the selective elimination of radioactive cesium

**DOI:** 10.1038/srep37009

**Published:** 2016-11-15

**Authors:** Adavan Kiliyankil Vipin, Bunshi Fugetsu, Ichiro Sakata, Akira Isogai, Morinobu Endo, Mingda Li, Mildred S. Dresselhaus

**Affiliations:** 1 School of Engineering, The University of Tokyo, 7-3-1 Bunkyo-ku, Tokyo 113-8656, Japan; 2 Policy Alternatives Research Institute, The University of Tokyo, 7-3-1 Bunkyo-ku, Tokyo 113-0033, Japan; 3 School of Agriculture and Life Sciences, The University of Tokyo 1-1-1 Yayoi, Bunkyo-ku, Tokyo 113-8657, Japan; 4 Institute of Carbon Science and Technology, Shinshu University, 4-17-1 Wakasato, Nagano 380-8553, Japan; 5 Department of Mechanical Engineering, Massachusetts Institute of Technology, 77 Massachusetts Avenue, Cambridge, MA 02139, USA; 6 Department of Physics and Department of Electrical Engineering and Computer Science, Massachusetts Institute of Technology, 77 Massachusetts Avenue, Cambridge, MA 02139, USA

## Abstract

On 11 March 2011, the day of the unforgettable disaster of the 9 magnitude Tohoku earthquake and quickly followed by the devastating Tsunami, a damageable amount of radionuclides had dispersed from the Fukushima Daiichi’s damaged nuclear reactors. Decontamination of the dispersed radionuclides from seawater and soil, due to the huge amounts of coexisting ions with competitive functionalities, has been the topmost difficulty. Ferric hexacyanoferrate, also known as Prussian blue (PB), has been the most powerful material for selectively trapping the radioactive cesium ions; its high tendency to form stable colloids in water, however, has made PB to be impossible for the open-field radioactive cesium decontamination applications. A nano/nano combinatorial approach, as is described in this study, has provided an ultimate solution to this intrinsic colloid formation difficulty of PB. Cellulose nanofibers (CNF) were used to immobilize PB via the creation of CNF-backboned PB. The CNF-backboned PB (CNF/PB) was found to be highly tolerant to water and moreover, it gave a 139 mg/g capability and a million (10^6^) order of magnitude distribution coefficient (K_d_) for absorbing of the radioactive cesium ion. Field studies on soil and seawater decontaminations in Fukushima gave satisfactory results, demonstrating high capabilities of CNF/PB for practical applications.

On 11 March 2011, the massive earthquake and tsunami struck the northeast coast of Japan, and led to a series of accidents at the Fukushima Daichi nuclear power plant and resulted in both the subsequent release of a huge quantity of radioactive isotopes into the atmosphere and thousands of tons of water have been catastrophically contaminated^1^. Even though five years have passed since the accident, the aftermath of the accident is still having a terrific impact not only on Japan but all over the world as well. The radioactive fallout mainly consisted of iodine-131, cesium-137, cesium-134, cesium-136, strontium-90, barium-140, zirconium-96 etc^2^. Out of the various species cited above, the two radioactive cesium isotopes contribute the most severe environmental impact, since they are both volatile fission products with a long half-life (Cs-137, T1/2 = 30 years and Cs-134, T1/2 = 2.06 years), and a high fission yield, that is easy to spread and to move over long distances into the external environment. Cesium mimics biologically important potassium and becomes distributed widely throughout the human body, thereby leading to high a level of intracellular accumulation. Radioactive cesium undergoes radioactive decay with the emission of strong beta and gamma radiation, which causes severe health effects^3^,^4^,^5^.

Scientists have already reported the use of inorganic ion exchangers such as zeolites, clay minerals, metal oxides and metal hexacyanoferrates, to deal with the radioactive cesium contamination^6^,^7^,^8^. The Fukushima Daiichi nuclear power stations are equipped with systems constructed with zeolite as the absorbing material, and these are under maximum operation for eliminating radioactive elements from high-level radioactive water. However, currently, there are no systems capable of eliminating the radioactive elements from the low-level radioactive water and soil. In fact, in March, 2011, after the earthquake and tsunami, over 10,000 tons of low-level radioactive water had been dumped from the storage tanks into the ocean to address the more critical task of decontaminating the high-level radioactive water^9^. The major difficulty which has been encountered in treating the low-level radioactive water and soil is that the radioactive elements are very low in concentration, compared to the co-existing abundant ions, sodium and potassium, especially for seawater; thus, the chosen adsorbing materials (adsorbents) must be highly selective for the targeted radioactive species. Prussian blue (ferric hexacyanoferrate), one of the first, man-made coordination compounds, widely known as “the blue pigment”, has been used for decontamination. PB is a mixed valence species with a basic cubic structure consisting of altering high spin iron (3^+^) ligated octahedrally by six nitrogen atoms and low spin iron (2^+^) is ligated by six carbon atoms of cyanide in a face centered cubic lattice to form ferricferrocyanide^10^,^11^. PB was famous for its zeolitic character, and acts as a molecular sieve and/or ion exchanger, because of the open structure of its crystal lattice and thereby is capable of hosting small molecules (water) and ions (Cs^+^)^12^. Therefore PB has long been considered as a potential adsorbent for radioactive cesium^13^. However, due to its intrinsic property of forming stable colloids in water, small sized (nano-sized) PB particles (thereby having a larger surface area and a higher adsorbing capacity) can easily contaminate water. For this reason, practical applications have long been limited to medical and/or pharmaceutical treatments^4^,^14^. Thus innovative immobilization without losing value is highly desired for the present applications^11^. Note that some PB analogs, like cobalt ferrocyanide^15^,^16^ and copper ferrocyanide^12^, are highly water-insoluble; these analog could be used directly as adsorbents for radioactive cesium eliminations. However, these analogs are costly compared to PB.

In this study, PB nanoparticles with cellulose nano-fiber (CNF) as backbones were synthesized via a nano/nano combination approach^17^. “The Great Wave off Kanagawa” by Hokusai, a famous artwork which makes extensive use of PB provided hints for making an effective CNF/PB complex. Moreover CNF has been used as a templating material for several nanoparticles, such as silver, gold, silicon, titaniatin oxide, ferrite etc^17^,^18^,^19^,^20^,^21^,^22^,^23^. However, CNF has not been used as a template for hosting the inorganic coordination complex material. CNF, an ecofriendly material, light weight and yet much stronger than steel doesn’t really need to be manufactured; rather it can be ‘grown’ from purified plant material. CNF is a polysaccharide consisting of a linear chain of several hundreds to many thousands of β (1→4) linked D-glucose units and has hydroxyl groups that are accessible for chemical modification^17^,^18^,^24^,^25^. The strong chelating ability of the CNF biopolymer matrix leads to the development of a functionalized complex material^26^.

Encapsulating the CNF backboned PB in a biopolymer matrix can be suitable for treating low-level radioactive water and soil. The polymer matrix allows development of different shapes such as beads, spongy foam, membranes and fibers. Several polymers are available for immobilization, and the most common species are alginate, chitosan, polyurethane and polyvinyl alcohol^27^,^28^,^29^. In our study polyvinyl alcohol (PVA) and polyurethane (PUF) were selected as a potential immobilizer for practical application of the CNF/PB complex material using the form of highly porous sponges.

In this research we demonstrate that CNF can be used as a template for synthesizing water insoluble PB for the effective decontamination of radioactive cesium. The treatment of low-level radioactive water and soil can be achieved by encapsulating CNF/PB in PVA and PUF. The laboratory scale decontamination of radioactive cesium was further scaled up to the pilot scale, followed by field studies, conducted in Fukushima, Japan.

We expanded our research into the full scale land restoration by decontamination of the water from radioactive cesium. We confirmed the feasibility by a demonstration test of the cesium adsorption from radioactive contaminated soil on actual farmland of Fukushima prefecture. In order to improve the practical efficiency of the decontamination by use of sponges we used plant seeds during agricultural sponge production and formation. We found that the water absorbing action of plant roots enhance the absorption of cesium from the contaminated soil.

## Results and Discussions

**Figure 1:**
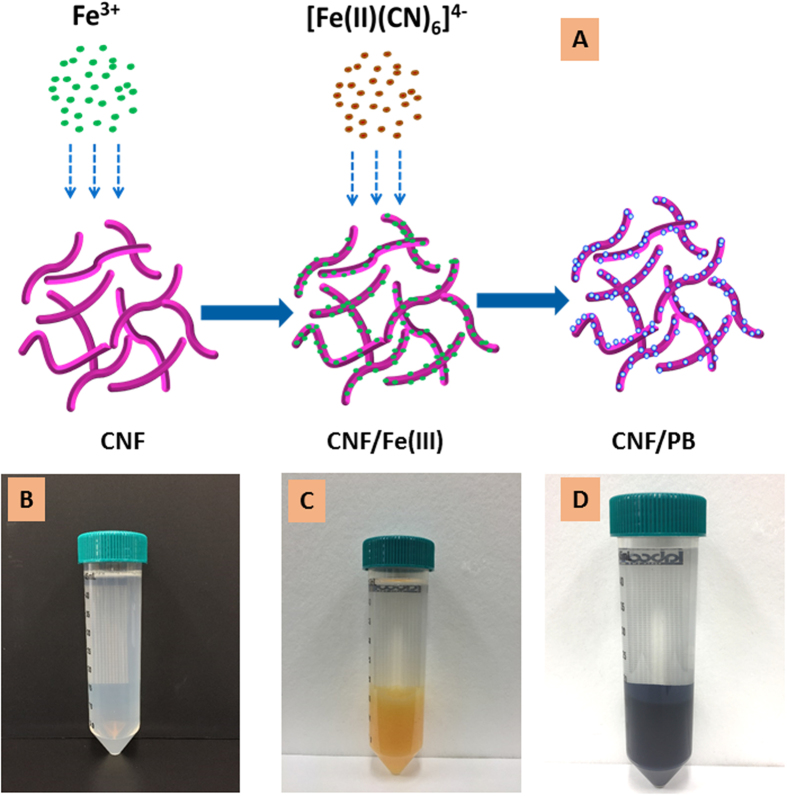
(**A**) A schematic representation of PB formation on CNF, (**B**) CNF dispersion in water, (**C**) CNF/Ferric complex in water and (**D**) CNF/PB complex in water.

**Figure 2:**
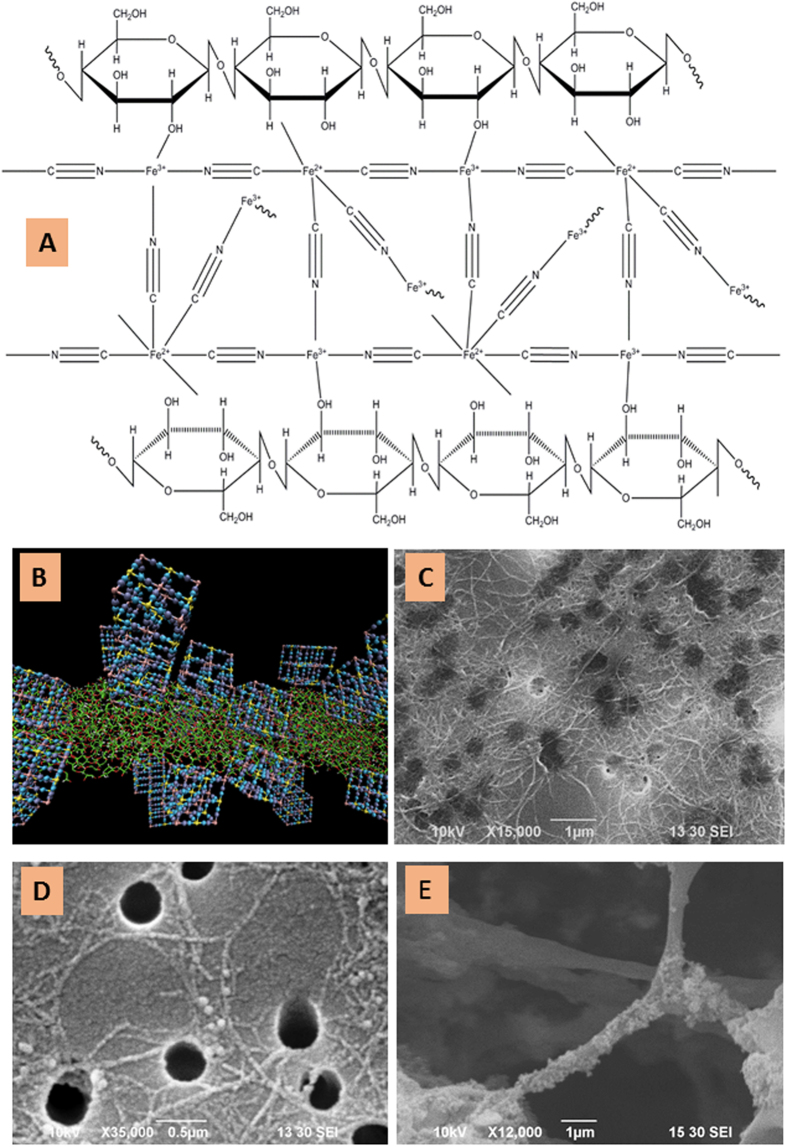
(**A**) Schematic representation of CNF/PB chelation, (**B**) a graphic outline of the CNF/PB complex. (**C**,**D**) SEM images of CNF, CNF/PB sample dried on the polycarbonate filter paper and (**E**) a freeze dried CNF/PB sample, respectively.

A schematic representation of the CNF/PB formation is depicted in Fig. 1A. The cellulose nanofiber (CNF; Fig. 1B) used throughout this study was 0.7–4 nm in diameter and 3–5 μm in length. The chemical structure of CNF/PB chelation is shown in Fig. 2A. The structural computer simulation of the CNF/PB complex is depicted in Fig. 2B. An SEM image of CNF is given in Fig. 2C. A typical AFM image for CNF is shown in the supporting information Figure S1A. In this study we propose the formation of nano-PB on CNF via surface coordination. β (1→4) Linked D-glucose units of CNF consist of abundant hydroxyl groups. The electron rich feature of the hydroxyl groups makes them suitable for active sites during the complexation reaction. When CNF was mixed with ferric chloride solution, the high spin ferric ion undergoes d^2^sp^3^ hybridization (Supporting information Figure S2) and makes some complexation with the hydroxyl group to form a CNF/Ferric (III) complex (Fig. 1C and supporting information Figure S1B). The coordination with an intermolecular water molecule present in CNF cannot be neglected. While adding hexacyanoferrate solution to the CNF/Ferric complex, a ligand exchange mechanism occurs resulting in the formation of a CNF-back-bonded PB (CNF/PB) complex^30^,^31^ (Fig. 1D). The SEM image of CNF/PB dried on polycarbonate filter paper (Fig. 2D) and freeze dried CNF/PB (Fig. 2E) confirmed the formation of nano-PB on the CNF.

We confirmed the advantageous properties of CNF over that of the normal sized cellulose obtained by preparing the PB complex with the normal sized cellulose as the backbones using the identical approach. The photograph in the supporting information Figure S3 confirmed that PB was hosted in a more stable manner with CNF than that obtained with the normal sized cellulose. This is an important advance because we observed a leakage of PB from the normal sized cellulose backboned PB.

**Figure 3:**
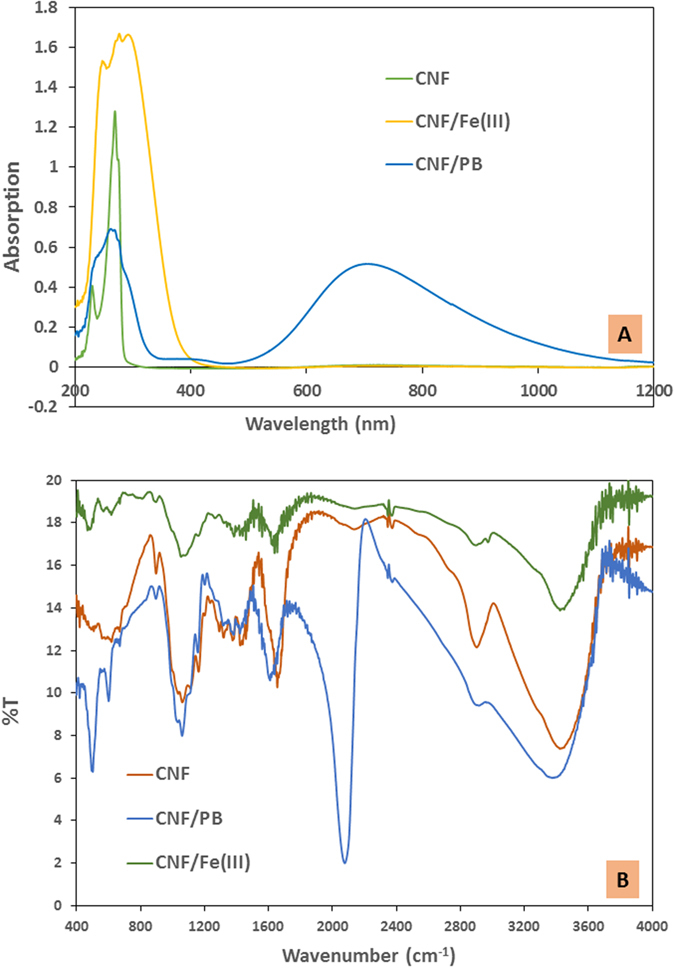
(**A**) UV-Vis spectra of CNF, CNF/Ferric (III), CNF/PB and (**B**) FT-IR spectra of CNF, CNF/Ferric(III) and CNF/PB, respectively.

Cellulose contains mostly saturated bonds and due to its phase heterogeneity in a suspension, it will strongly scatter visible light and absorb UV light (Fig. 3A), so that the characteristic peaks of the chemical changes were mostly located in the UV region of the UV-Vis spectrum. CNF exhibits two characteristic peaks, one is a strong and sharp peak at λ_max_ = 269 nm, and the other is a relatively low intensity peak at λ_max_ = 230 nm. The CNF peak at λ_max_ = 269 nm was present in both of the CNF/ferric (III) and the CNF/PB absorption spectra. In the CNF/Ferric (III) spectra a sharp new peak was identified at λ_max_ = 275 nm thereby confirming the complexation between CNF and ferric ion (III) which is attributed to a π-π* transition of the resonance structure of CNF. The broad band with λ_max_ at 690 nm due to the inter-metal charge transfer from Fe^2+^ to Fe^3+^ reflects the confirmation of PB formation^26^,^32^,^33^,^34^, shown by the blue curve in Fig. 3A.

FT-IR is a laboratory tool used to investigate the chemical variations during complexation reactions with CNF. The FT-IR spectrum of the original CNF, CNF-ferric (III) complex and the final CNF/PB complex are shown in Fig. 3B. The characteristic FT-IR peaks at 1029 cm^−1^ (C-H bending), 3200–3500 cm^−1^ (O-H stretching), 2850–3000 cm^−1^ (C-H stretching), 1609–1655 cm^−1^ (O-H bending) are clearly visible. The band at 1059–1162 cm^−1^ corresponds to C-O and C-O-C stretching bond in the CNF. Peaks at 1631 cm^−1^ and 890 cm^−1^ corresponds to the C=O stretching and glycoside linkage in cellulose respectively^21^,^35^,^36^,^37^. The characteristic sharp peak of the Fe-CN stretching vibration at 2072 cm^−1^ in the CNF/PB FT-IR spectrum confirms the presence of PB. The peaks at 501 cm^−1^ and 594 cm^−1^ correspond to the metal-ligand bonds in the PB^38^,^39^,^40^,^41^,^42^. Note here that the C-O and the C-O-C stretching bonds in the CNF remain almost the same in both the CNF-ferric (III) complex and the CNF/PB complex, indicating that the ring structure of the CNF did not change. Thus we could confirm that the chelation occurred only at the highly reactive primary hydroxyl groups and the secondary hydroxyl group remain unchanged^18^.

**Figure 4:**
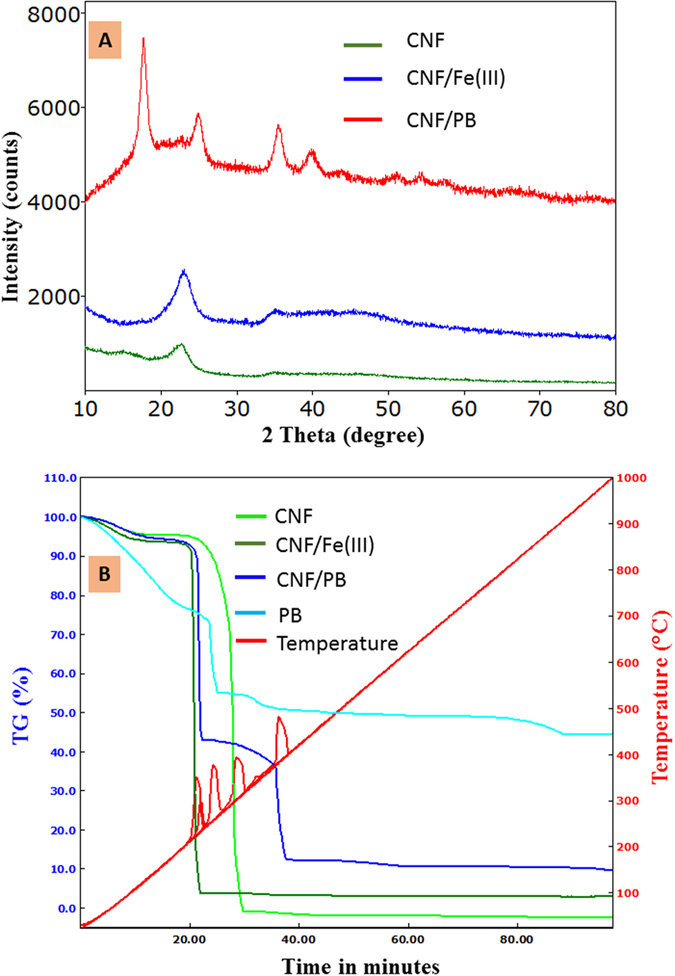
(**A**) XRD spectra of CNF, CNF/Ferric (III) and CNF/PB and (**B**) Thermogram (TG) of CNF, CNF/Ferric (III), CNF/PB, respectively.

The XRD pattern of CNF, CNF-ferric (III) and CNF/PB are as shown in Fig. 4A. The broad peak at 23.06° in CNF indicates the amorphous nature of CNF. The XRD pattern remained unchanged after the complexation of CNF with ferric (III) ions. The crystal structure of PB has been explained in the pioneering work by Keggin and Miles, who defined the unit cell as face centered cubic structure with space group Fm3 m. The characteristic peaks of the CNF/PB complex are located at 17.57°, 24.84°, 35.63°, 39.64° and 50.8° corresponding to the (200), (220), (400), (420) and (440) crystal planes, respectively. We observed that the amorphous peak of the CNF backbone was slightly broadened in the first two peaks of the CNF/PB XRD pattern. The XRD pattern thus confirms the formation of highly crystalline PB with space group Fm3 m on the CNF matrix^29^,^40^,^41^,^43^,^44^.

Thermogravimetric analysis (TGA) reveals that pure CNF decomposes completely without any residue (Fig. 4B). Remaining residues for the CNF-ferric (III) complex and CNF/PB were found to be at about 3% and 10%, respectively, which confirm the incorporation of ferric ion and PB with CNF. From the gravimetric analysis we confirmed that the CNF can hold 30% PB relative to its own weight. The weight loss below 150 °C was attributed to a loss of water by evaporation. The degradation of CNF starts from 190 °C and is a maximum at 304 °C, and is complete at 357 °C. But in the case of CNF-Ferric (III) and CNF/PB, the thermal degradation is slightly shifted to lower temperature, because of the loss of the primary hydroxyl group and the intermolecular water molecule during complexation^36^,^45^. The thermal degradation of CNF/PB has two major steps: Step 1 (145–265 °C) and Step 2 (265–435 °C). In Step1 the weight loss is mainly due to the CNF degradation and the release of the crystalline water molecule coordinated with PB crystals^29^,^43^,^46^,^47^. The weight loss in Step 2 is mainly due to the decomposition of the cyanide group. Further weight reduction occurs by the evolution of oxides of nitrogen and carbon. The thermal decomposition of both the CNF-Ferric (III) and CNF/PB complex end up with Fe_2_O_3_ as the final solid remaining. We observed that all the major decomposition that occurred is exothermic in nature^20^,^21^,^22^.

**Figure 5:**
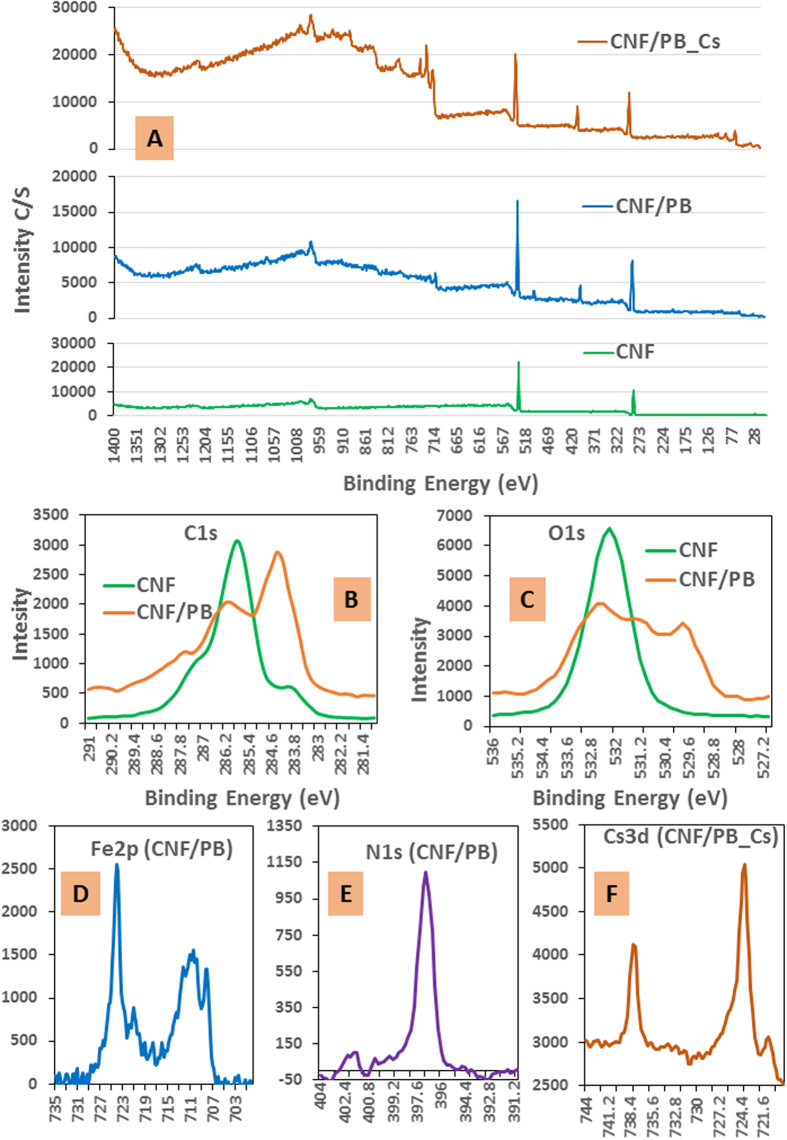
(**A**) Low resolution XPS spectra of CNF, CNF/PB and CNF/PB after Cs adsorption; (**B**) high resolution spectra of C1s shown for CNF and CNF/PB, (**C**) O1s spectra shown for CNF and CNF/PB, (**D**) Fe2p of CNF/PB, (**E**) N1s of CNF/PB and F) Cs3d of CNF/PB after Cs adsorption.

XPS is a useful and sensitive tool to investigate the surface chemical modification as shown in Fig. 5A–F. Figure 5A shows the low resolution XPS spectra of CNF, CNF/PB and the CNF/PB after Cs adsorption, respectively. The high resolution spectra of C1s (Fig. 5B) gives four deconvolution peaks and each corresponds to carbon without an oxygen bond (C-1), carbon with one oxygen bond (C-2), carbon with two oxygen bonds (C-3) and carbon with three oxygen bonds (C-4). In the XPS spectra of CNF, the strong C1s peak at 286.1 eV was mainly related to the C-2 carbon. But, in CNF/PB spectrum the strong C1s peak at 284.5 eV was related to the C-1 carbon, while the C-2 peak was relatively weak^21^,^37^,^48^,^49^. This change confirms that the primary hydroxyl groups were involved in the complexation. Also, the calculation of the atomic ratio of O/C (Fig. 5B,C) shows that the CNF/PB nano/nano combination decreased the ratio by about 0.37^50^. The high resolution XPS spectra of Fe 2p (Fig. 5D) showed two characteristic peaks at the binding energies 710.3 eV and 724.1 eV of Fe2p_3/2_ and Fe2p_1/2_ respectively, which originated from the presence of the ferric ion. The satellite peak obtained between these two peaks at 718.8 eV is clearly distinguishable and does not overlap either peaks was the interpreted as the electronic structure of the ferric ion. The sharp peak at 708.3 eV and the small peak at 721.8 eV in Fig. 5D can be assigned to the Fe 2p peak of the ferrous ion in the Ferro cyanide^33^,^51^. The sharp peak of the N1s of nitrogen (Fig. 5E) can be resolved into three components, a strong peak at 397.1 eV and weak peaks at binding energies 399.5 eV and 401.7 eV. These observations signify the presence of C-N ([Fe (CN)_6_]) in the CNF/PB complex. On the basis of the higher resolution spectral analysis of the N1s and Fe 2p spectra we conclude that the PB particle was successfully identified even with CNF as its backbone^52^,^53^,^54^. The caging of the cesium ion in CNF/PB is mainly due to an ion-exchange between the water molecules and/or the monovalent cations from the crystal lattice of PB. In this experiment, Na_4_Fe (CN)_6_ was an ingredient for the production of PB; this leads to the generation of PB with sodium ions being caged into the crystal lattice. Cesium ion can readily penetrate into the PB crystal lattice and can easily displace sodium ions from the lattice. These assumptions were clearly justified from the XPS analysis of the CNF/PB complex before and after the adsorption test. The typical peak of Na1s spectrum was clearly visible at a binding energy 1071 eV in the XPS spectrum of the CNF/PB complex before the cesium adsorption. The spectrum of the CNF/PB powder after the cesium adsorption reveals that each sodium ion was replaced by a cesium ion. Therefore that the characteristic spectrum Cs 3d (Fig. 5F) was found at 737.8 eV (Cs 3d3) and 723.9 eV (Cs 3d5) and the Na1s peak was absent.

The nitrogen adsorption/desorption isotherm of the CNF/PB complex was found to follow a type IV isotherm (Supporting information Figure S4). The BET surface area and the Langmuir surface area (monolayer) of the CNF/PB complex were 161.9 m^2^/g was 149.4 m^2^/g, respectively. EDS for CNF/PB doubly confirmed the content of Ferric obtained in TGA. Besides, EDS mapping could give the information of distribution of iron on CNF. The EDS mapping of CNF/PB is given in the supporting information Figure S5.

Equilibrium isotherms are the most widely accepted tool providing information about the maximum absorption capacity, the sorption mechanism, surface properties, and the affinity of the sorbent. In this study, we considered the Langmuir and Freundlich isotherm models to analyze the adsorption behavior. In our experiment, the Langmuir R^2^ value was higher than the Freundlich isotherm, indicating that the adsorption mainly follows the Langmuir model (supporting information Figure S6). The maximum adsorption capacity was calculated from the slope and the intercept values of the Langmuir plot. The maximum cesium adsorption capacity of the CNF/PB complex was 139 mg/g where PB was nearly 30 wt%, so that the adsorption capacity based on PB was ~463 mg/g, which is very close to the existing adsorption capacity as given in previous studies^4^,^28^,^29^. Surprisingly the adsorption capacity was tremendous and sufficient to solve the high level of cesium contamination in spent fuel tank. Since the PB was stabilized by CNF, we could use the complex material directly in treating contaminated water. Moreover, the thermal stability was confirmed by thermogravimetric analysis.

**Figure 6:**
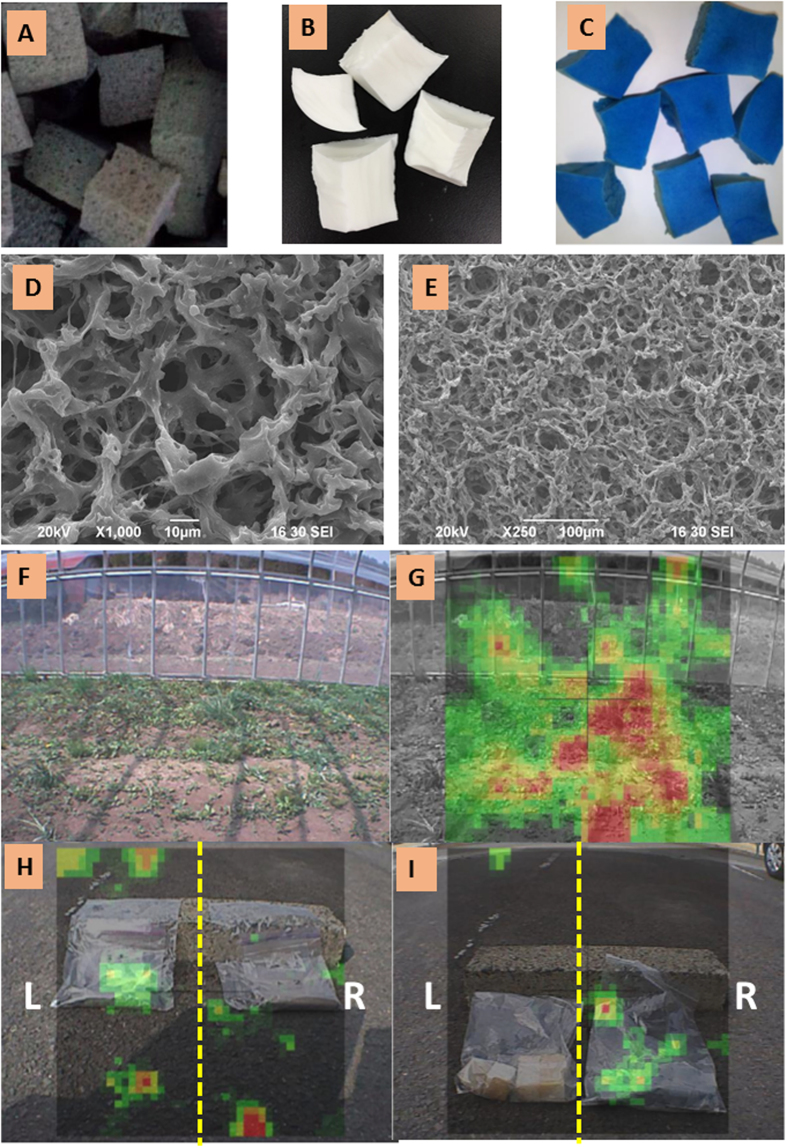
(**A**) CNF/PB/PUF sponge, (**B**) blank PVA sponge, (**C**) CNF/PB/PVA sponge; SEM images of sponges are shown in (**D**) at high magnification (1000X) and (**E**) low magnification (250X). (**F**) Photograph of the subject area for decontamination; (**G**) radiation camera images of soil radiation before decontamination, (**H**) soil radiation after decontamination by the pure PUF sponge (L) and the CNF/PB/PUF sponge (R) and (**I**) sponge radiation after Cs adsorption by the pure PUF sponge (L) and CNF/PB/PUF sponge (R).

Our research was further expanded to the remediation of medium and low levels of cesium contamination. The overcapacity was the major problem when dealing with low levels of radioactive pollution. In order to face this issue, we used a CNF/PB based spongiform adsorbent, in which we could control the amount of CNF/PB, depending on the radioactive contamination level. The preparation of PUF sponge was very easy and is the most widely used spongiform (Fig. 6A). But in our experience the CNF/PB holding capacity in PUF was limited to a maximum value of 5 wt%. However, the 5 wt% capacity was more than enough to deal with the low level of radiation in the real environmental situation. The maximum cesium adsorption capacity of CNF/PB/PUF ranges from 0.5–10 mg/g depending on the amount of the CNF/PB complex in the sponge. We used PVA to incorporate the CNF/PB complex with more than 5 wt% in the spongiform adsorbent. The photographs of the pure PVA sponge and the CNF/PB/PVA sponge are given in Fig. 6B,C, respectively. The porosity of the CNF/PB/PVA spongiform is observed by SEM and the images are shown in Fig. 6D,E) Photograph of the subject area for decontamination and the radiation camera images of soil radiation before decontamination are shown in Fig. 6F,G, respectively. The combined radioactivity (Cs-134 and Cs-137) at the farmland before decontamination was 27,146 Bq/Kg (n = 5). After restoration work, the combined radioactivity declined to half of its initial value, 14,816 Bq/Kg (n = 5). The changes in the amount of radiation was observed by a radiation camera and the images are presented in Fig. 6H,I.

The higher encapsulation efficiency of PVA due to the long polymeric structure compares to polyurethane allows PVA to hold up to 30 wt% of CNF/PB in the sponge without a leak of CNF/PB. The stable CNF/PB/PVA sponge could perform a maximum adsorption capacity ranging from 10–28.4 mg/g depending on the amount of CNF/PB complex in the sponge. The maximum adsorption capacity can be calculated from the slope and intercept values of the Langmuir plot, as given in the supporting information in Figure S7. The adsorption of the cesium ion by the spongiform adsorbent at various concentrations is depicted in the supporting information in Figure S8. The CNF/PB/PVA sponge, due to its higher PB content and the higher density (0.121 g/cm^3^), was prepared for the higher level cesium eliminations. The CNF/PB/PUF, on the other hand, due to its lower PB content and the lower density (0.038 g/cm^3^), was desirable for the low level radioactive contamination eliminations.

**Figure 7:**
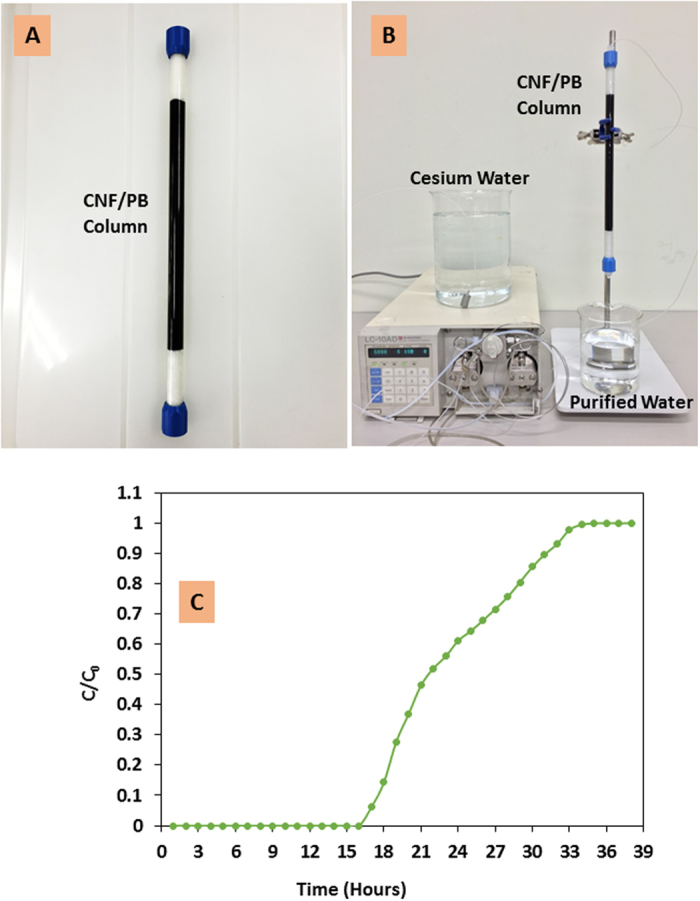
(**A**) CNF/PB fixed bed column, (**B**) laboratory set up of a pilot scale column adsorption test and (**C**) breakthrough curve for the CNF/PB column for Cs ion adsorption.

A pilot- scale, column-type device was established using CNF/PB (Fig. 7A,B); cesium was not detected until 16.5 hours of operation at which time a total of 2.5 liters of the 151 ppm Cs ion solution has passed through the column; the total adsorption capacity at the point of breakthrough (Fig. 7C) was found to be 70.5 mg/g (a total 5.3 g of CNF/PB was packed in the column).

**Figure 8:**
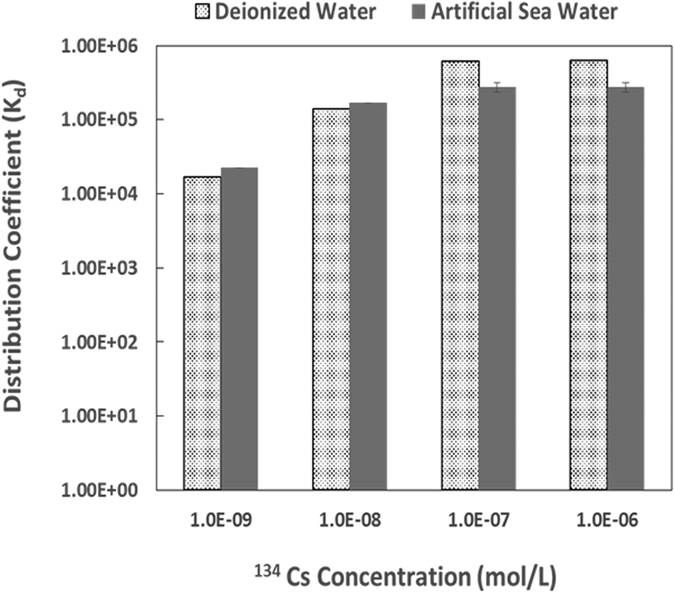
Cs-134 distribution coefficient toward the CNF/PB/PVA sponge; both deionized water and artificial seawater samples were examined.

From the Cs-134 elimination test, the distribution coefficient, K_d_, was found to be at 10^4^ ml/g levels for samples of lower Cs-134 concentrations; while the K_d_ value increased to 10^5^ ml/g levels for samples of higher Cs-134 (Fig. 8). The distribution coefficient for Cs-134 absorption in the seawater is better than that in pure water at lower cesium ion concentrations; while it declined slightly for the seawater samples at higher cesium ion concentrations. The ultrahigh concentrations of the competitive ions (Na^+^, K^+^, Mg^2+^, etc.) which existed in the seawater have slightly decreased the efficiency of PB to absorb cesium ions. The Cs-134 elimination efficiency of the CNF/PB/PVA sponge from deionized water and artificial seawater were 99.2% and 99.9% respectively. The cesium ion was selectively retained by the CNF-backboned PB nanoparticles from the other competitive ions with the so-called size-based affinity interaction acting as the key reaction mechanism.

After the Fukushima disaster, this is to our knowledge, the first time to achieve a successful decontamination system. The successful growth of plants and the seed germination from the sponge in an active field test is given in the supporting information in Figure S9. The decontamination by the CNF/PB/PUF sponge without seeds in the vinyl house after four weeks was 21.6% with average adsorption of 535 Bq/Kg and that of the CNF/PB/PVA sponge after one week was 19% with an average adsorption 240 Bq/Kg. The average adsorption of the CNF/PB/PUF sponge in open land was 917 Bq/Kg and that of the CNF/PB/PVA sponge was 141 Bq/Kg. The adsorption of the radioactive cesium from soil by the CNF/PB/PUF sponge with seeds was surprisingly high. The average adsorption at the vinyl greenhouse and open land were 917 Bq/Kg and 1043 Bq/Kg, respectively. The above results confirmed the cooperation of plants to enhance the radiation adsorption. The average absorption with seeds was higher than that without seeds. Long-term studies on exploring information about whether the seeds themselves can absorb cesium ions during growth has been undertaken by our groups. If seeds themselves can absorb cesium ions, then a control experiment with seeds in the soil shall be introduced to determine how much enhanced absorption is due to the seed growth, and then the rest of the enhancement can be attributed to the absorption of more cesium ions by the CNF/PB sponge assisted by the water absorbing action of the plant roots.

The mechanism of cesium adsorption was explained on the basis of isothermal studies. The Langmuir isotherm is the most widely used isotherm^55^ that assumes homogeneous monolayer adsorption on well-defined sites, which are identical and equivalent. Each of the definite localized sites holds one molecule and possesses an equal enthalpy and sorption energy. Equilibrium describes the point beyond which no further adsorption takes place. Neither lateral interaction between the adsorbed molecules nor transmigration of the adsorbate in the plane of the surface take place at once equilibrium is established.

## Conclusions

Cellulose nanofiber (CNF) backboned Prussian blue (PB) is found to be highly tolerant to water. This unique water-insoluble property is highly desirable for radioactive cesium elimination. Considerable complexation between CNF and PB nanoparticles is the key interaction occurring for the CNF/PB complex formation. Capabilities and behaviors of cesium sorption by the CNF-backboned PB nanoparticles were evaluated via a batch adsorption and a breakthrough approach. The performance was tested with low, medium and high levels of cesium concentration. The adsorption follows a Langmuir isotherm with a theoretical cesium capacity of 139 mg/g for CNF/PB complex powder and 463 mg/g for PB. The absolute safe adsorption capacity is established in a fixed bed column was 70 mg/g of the CNF/PB complex powder. The CNF/PB complex can be further encapsulated into a PUF and PVA sponge to overcome radiation over-loading by controlling the absorption based on the level of radioactive cesium. We confirmed the feasibility by the demonstration test of the cesium adsorption from radioactive contaminated soil in the farmland of Fukushima Prefecture by using the CNF/PB encapsulated spongiform adsorbents. The combined radioactivity (Cs-134 and Cs-137) at the farmland declined to half of its initial value within a month. Thus, this new class of the “chelate-like”, water-insoluble, three-dimensional porous nanostructure can open up new possibilities for effectively eliminating radioactive cesium from contaminated waters and soil.

## Methods

### Materials

Ferric chloride (FeCl_3_.6H_2_O), sodium ferrocyanide (Na_4_[Fe(CN)_6_].10H_2_O), cesium chloride (CsCl), polyvinyl alcohol (polymerization degree 2000), corn starch, potato starch, formaldehyde (HCHO, 36–38%), sulfuric acid (H_2_SO_4_, 95%) were purchased from the Wako Pure Chemicals company (Osaka, Japan). Cellulose nano-fiber (1.0 wt%) produced based on “Isogai method” was purchased from Nihon Seishi.

### Preparation of cellulose nano-fiber backboned Prussian blue (CNF/PB)

120 g of 1 wt% CNF dispersed in 900 mL deionized water and was slowly mixed with 25 mL of 0.5 M ferric chloride solution in a beaker by mechanical stirring. The mixture was kept overnight in an oven at 60 °C to complete the chelating reaction (immobilizing Fe(III) ions onto CNF based on a chelating interaction). The mixture was then allowed to cool down to room temperature and was slowly added to 25 mL 0.125 M sodium ferrocyanide solution under overnight continuous stirring. The resultant CNF/PB was insoluble and completely settled down in water so that it was very easy to wash with deionized water (the settling property of CNF/PB is shown in the supporting information Figure S10). After washing several times with deionized water, the CNF/PB was freeze dried. The freeze dried sample can later be washed again to ensure purity and further air drying if is required, as shown in supporting information Figure S11.

### Preparation of CNF/PB polyurethane sponge (CNF/PB/PUF)

The CNF/PB solution was mixed with polyurethane prepolymer (NB-9000B) to produce the spongiform. NB-9000B is derived from poly(oxy C2-4 alkylene) diol and toluene diisocyanate and has three isocyanate functionalities capable of forming spongiform when combined with water molecules. Pluronic L-62, a tri-block copolymer, was used to strengthen the cell walls of the polyurethane foam. The ratio of CNF/PB, NB-9000B, Pluronic-L-62 and water was optimized to get a high quality adsorbent^28^. The composite sponge was dried at 70 °C for 48 hours and washed with deionized water.

### Preparation of CNF/PB polyvinyl alcohol sponge (CNF/PB/PVA)

20 g of PVA dissolved in 200 mL deionized water obtained by heating under a water bath at 90 °C for 3 hours with constant stirring at 150 rpm. 10 g of each starch was agitated in 30 mL deionized water and added to the homogeneous solution of PVA. This solution was then heated under the water bath at 90 °C for 45 minutes with constant stirring at 200 rpm to get a clear solution. Next we prepare a homogeneous colloidal solution of CNF/PB (0.5 g–10 g) in a beaker with mechanical stirring. This colloidal mixture was then added to the above PVA starch mixture. The solution was then heated under a water bath at 90 °C with constant stirring at 300 rpm until the total volume became 250 mL. The mixture was then allowed to cool down to 20 °C using ice water and a mixture of formaldehyde (25 mL), and sulfuric acid (25 mL) was added drop by drop with constant stirring at 400 rpm. The colloidal solution was then kept overnight in hot water at 55 °C. The developed CNF/PB/PVA sponge was collected and washed thoroughly with deionized water and used.

### Material characterization

The morphology of the samples was observed by JEOL JSM 6390 Scanning electron microscopy (SEM). A thermal decomposition study was conducted over the temperature range of 30–1000 °C using an SII EXSTAR 6000 TG/DTA analyzer. The UV-visible and FT-IR spectra were acquired with a JASCO V-570 UV/VIS/NIR spectrometer and JASCO FT/IR-460 instrument, respectively. The X-ray diffraction (XRD) measurement was conducted with the help of a Rigaku SuperLab (Kα1) diffraction apparatus using Cu-Kα radiation. The X-ray photoelectron Spectroscopic analysis (XPS) was done with a multifunctional Scanning X-ray Photoelectron Spectrometer PHI5000 VersaProbe (UILVAC-PHI). The BET (Brunauer, Emmett and Teller) surface area was obtained from nitrogen adsorption/desorption isotherm using a Beckman Coulter SA™ 3100 analyzer. The elemental distribution was analyzed by using the Energy dispersive X-ray spectroscopy (EDS) method.

### Adsorption experiments

#### Batch Adsorption

CNF/PB powder and sponges were air dried for 20 min at 80 °C. The known weight of the adsorbent was added into cesium solutions at different concentrations (50 ppb to 250 ppm) in a sealable plastic tube. The solution was shaken on a vortex shaker at room temperature. The samples were collected at different time intervals and the remaining cesium ions left in solution were determined by an inductively coupled plasma-atomic emission spectrometer and an Atomic Absorption Spectroscope^29^. The adsorption studies included the determination of important adsorption parameters, such as the adsorption capacity (q), and these parameters can be calculated by using the following equation^56^,^57^:


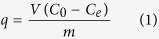


where *C*_0_ and *C*_*e*_ are the initial and final concentrations (mg/L) of the adsorbate ions in the aqueous solution, respectively, V is the volume of the solution (L), and m is the weight of the adsorbent (g).

The sorption of each sample was tested in triplicate and averages were used to evaluate both the sorption parameters and the accuracy of the measurement. In order to understand the sorption behavior and mechanism, isotherm models are very useful. In this study the Langmuir and Freundlich models were used.

### Fixed bed column adsorption

We undertook a series of continuous fixed bed column analyses to investigate the practical applicability of the CNF/PB adsorbent. A fixed bed system was prepared with a chromatographic glass column of length 30 cm and internal diameter 1.0 cm. The column was filled with 15 mL (5.3 g) CNF/PB complex (Fig. 7A). The artificial Cs solution (deionized water contains 151 ppm cesium chloride) has been pumped through the column at a constant flow rate 2.5 mL/min (Fig. 7B). The column study was specifically focused on the practical feasibility of large-scale treatment of cesium contaminated water^58^,^59^.

### Removal of radioactive cesium from water

A test solution was prepared by adding a radioactive Cs-134 tracer and stable cesium into pure water or artificial sea water. In this study a liquid radioactivity tracer was used to evaluate the distribution coefficient in the ICP-MS analysis in a non-low density area. 30 mL solution was taken in a sealable tube and shaken with 0.15 g CNF/PB/PVA sponge at 80 rpm for 7 days. Using a NaI scintillation counter, the radioactivity count rate before and after the adsorbing [cpm (count/min)] were respectively measured to calculate the partition coefficient by the following equation^27^.


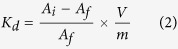


were, A_i_ and A_f_ are the radioactivity count rates before and after the adsorbing the radioactive material [cpm (count/min)], V is the volume of the solution (mL), and m is the weight of the adsorbent (g). Also, simulated radioactive water samples were prepared by adding cesium-137 into deionized water and seawater. We chose a cesium- 137 activity of 1.50 Bq/mL. The simulated water samples (10 mL) were taken up into the spongiform absorbents (approximately 0.25 g of each adsorbent). After about 10-h of water/spongy contacting, the water was squeezed out from the spongy sample and the residual radioactivity in the water was analyzed.

### Removal of radioactive cesium from the soil (Fukushima active site)

CNF/PB encapsulated PUF and PVA sponges blended with plant seeds were made on an industrial scale for the field test. We cut the sponges into cubes (3 cm × 3 cm × 3 cm) and buried these cubes into to the contaminated soil (from the surface down to 8 cm). The target areas were 20 m × 4 m vinyl green house and 10 m × 8 m open land (supporting information Figure S12). We conducted decontamination test for three weeks with the PUF sponge, and for one week with PVA sponges. The practical set up of decontamination, using the composite sponge with seeds is shown in the supporting information Figure S13. The decontamination sponge (cesium adsorption) can be easily be embedded in a desirable place in the soil without damaging the blade of the tractor. The plant that has been grown from seeds in the decontamination sponge can be easily recovered, along with the roots. The collected and stored decontamination sponge volume can be decreased by compression and eventually high temperature incineration is also possible for disposal. The growth of the plants could be different, depending on the species of the type of plants to be included in the decontamination sponge. The new method of decontamination is almost maintenance-free and easy.

## Additional Information

**How to cite this article**: Vipin, A. K. *et al*. Cellulose nanofiber backboned Prussian blue nanoparticles as powerful adsorbents for the selective elimination of radioactive cesium. *Sci. Rep.* **6**, 37009; doi: 10.1038/srep37009 (2016).

**Publisher’s note**: Springer Nature remains neutral with regard to jurisdictional claims in published maps and institutional affiliations.

## Supplementary Material

Supplementary Information
