# Supplementary material for: Cellulose nanofiber backboned Prussian blue nanoparticles as powerful adsorbents for the selective elimination of radioactive cesium
Source: Sci Rep. 2016 Nov 15;6:37009. doi: 10.1038/srep37009 (PMC5109467; doi:10.1038/srep37009)
Supplement: Supplementary Information [file srep37009-s1.pdf]

Supporting information

Cellulose nanofiber backboned Prussian blue nanoparticles as powerful adsorbents for the selective elimination of radioactive cesium

Adavan Kiliyankil Vipin[1*], Bunshi Fugetsu[2], Ichiro Sakata[1,2], Akira Isogai[3], Morinobu Endo[4], Mingda Li[5], Mildred S. Dresselhaus[6]

[1] School of Engineering, The University of Tokyo, Bunkyo-ku, Tokyo 113-0032, Japan

[2] Policy Alternative Research Institute, The University of Tokyo, Bunkyo-ku, Tokyo 113-0032, Japan

[3] School of Agriculture and Life Sciences, The University of Tokyo, Tokyo 113-8657, Japan

[4] Institute of Carbon Science and Technology, Shinshu University, 4-17-1 Wakasato, Nagano 380-8553, Japan

[5] Department of Mechanical Engineering, Massachusetts Institute of Technology, 77 Massachusetts Avenue, Cambridge, MA 02139, USA.

[6] Department of Physics and Department of Electrical Engineering and Computer Science, Massachusetts Institute of Technology, 77 Massachusetts Avenue, Cambridge, MA 02139, USA.

*Correspondence and request for materials should be addressed to A.K.V. (E-mail: vipin@ipr-ctr.t.u.-tokyo.ac.jp)

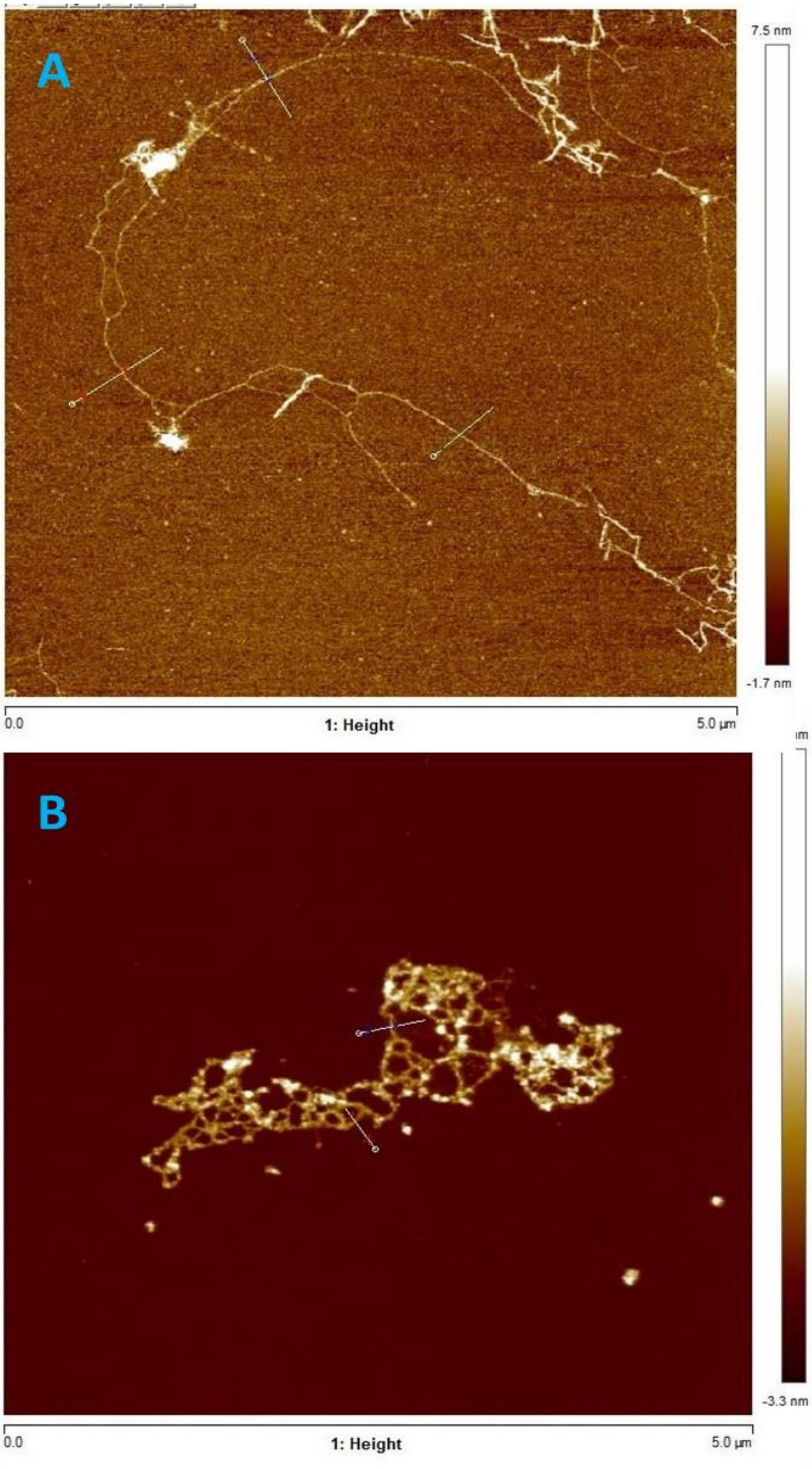

Figure S1. AFM images: A) CNF and B) CNF/Ferric(III).

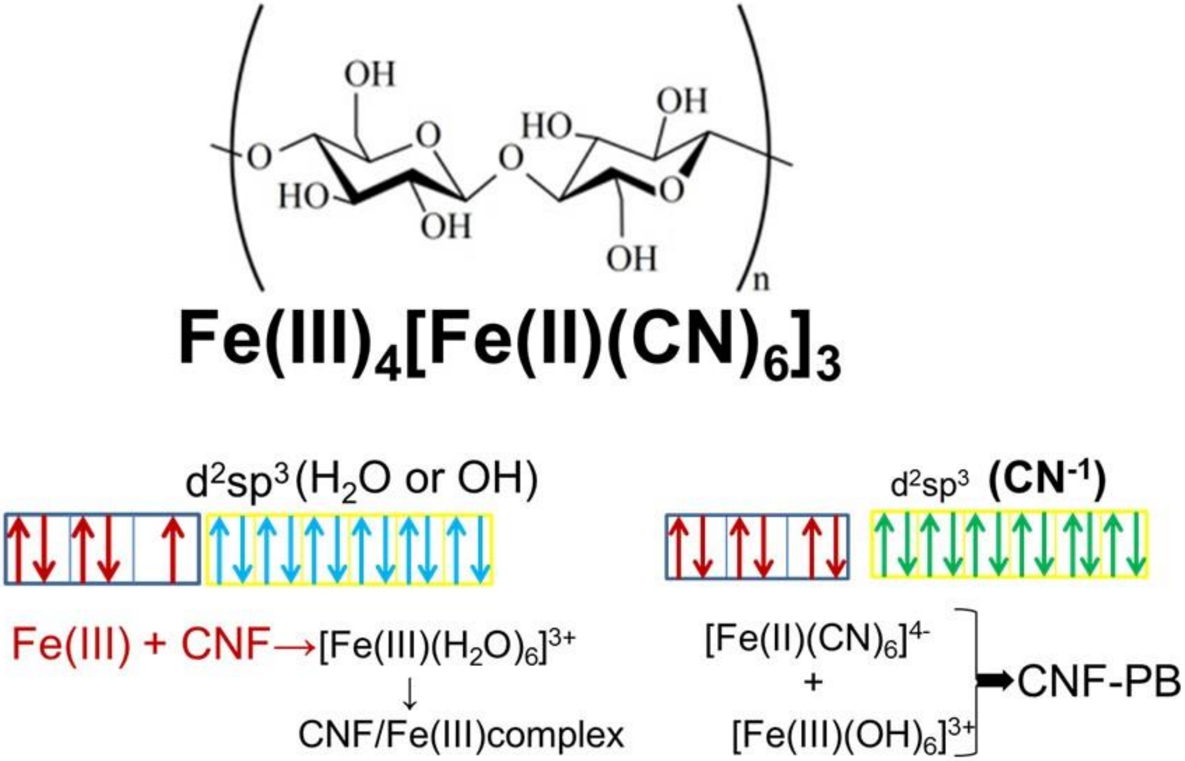

Figure S2. Formation of the chelated CNF/PB complex.

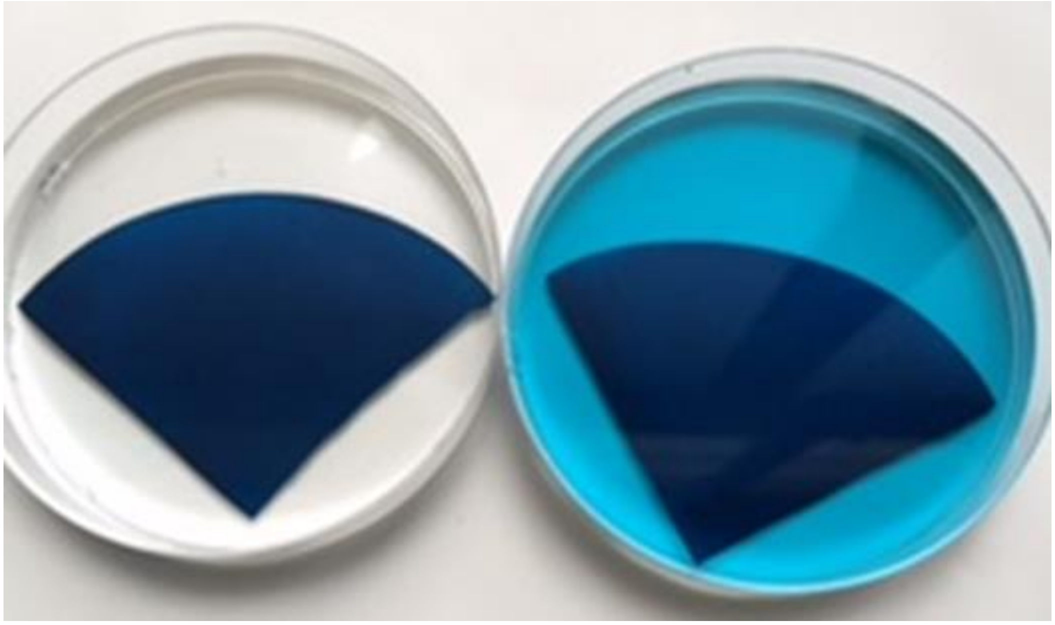

Figure S3. Photos of a CNF/PB sample (left) and a normal sized cellulose/PB sample (right) being immerged in tap water for two days.

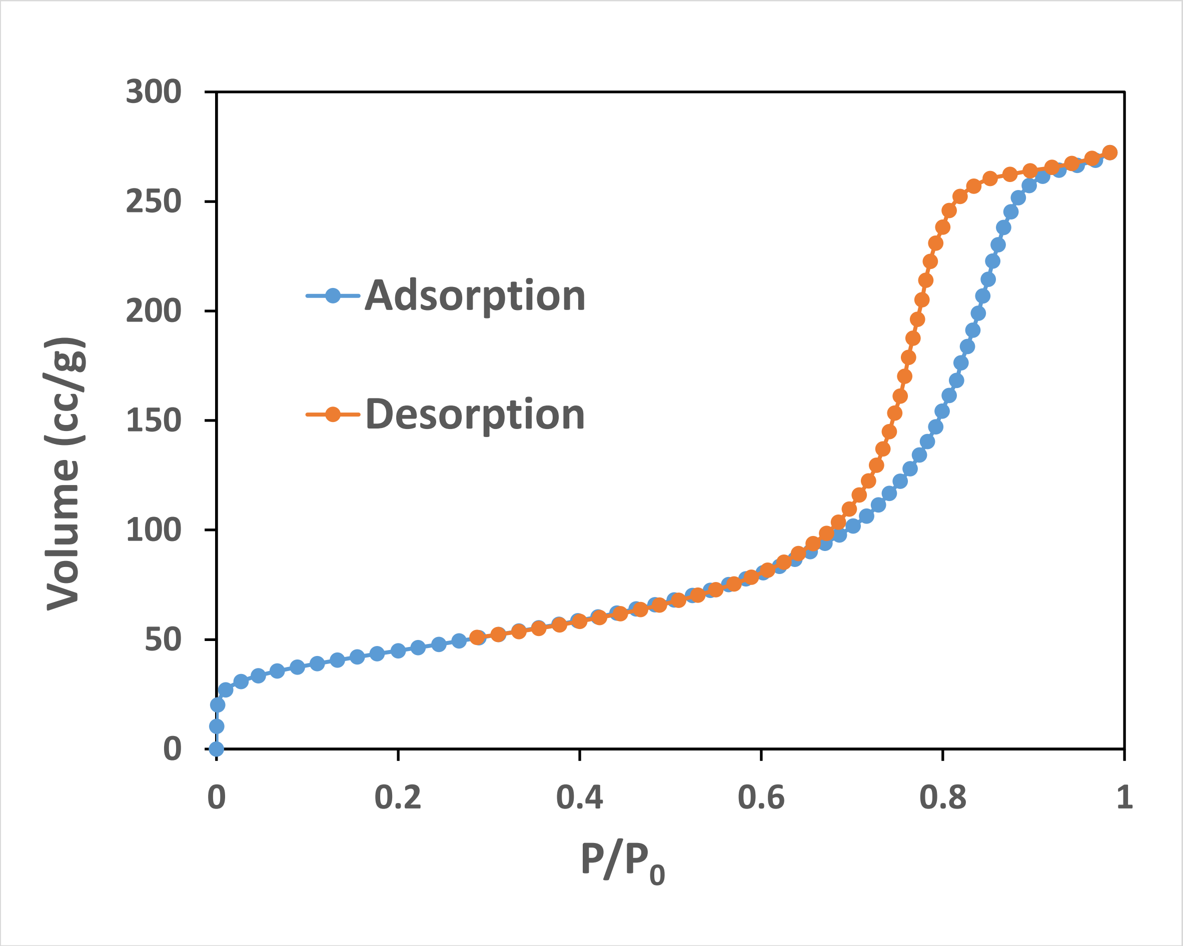

Figure S4. Nitrogen adsorption and desorption isotherms of a typical CNF/PB complex powder sample.

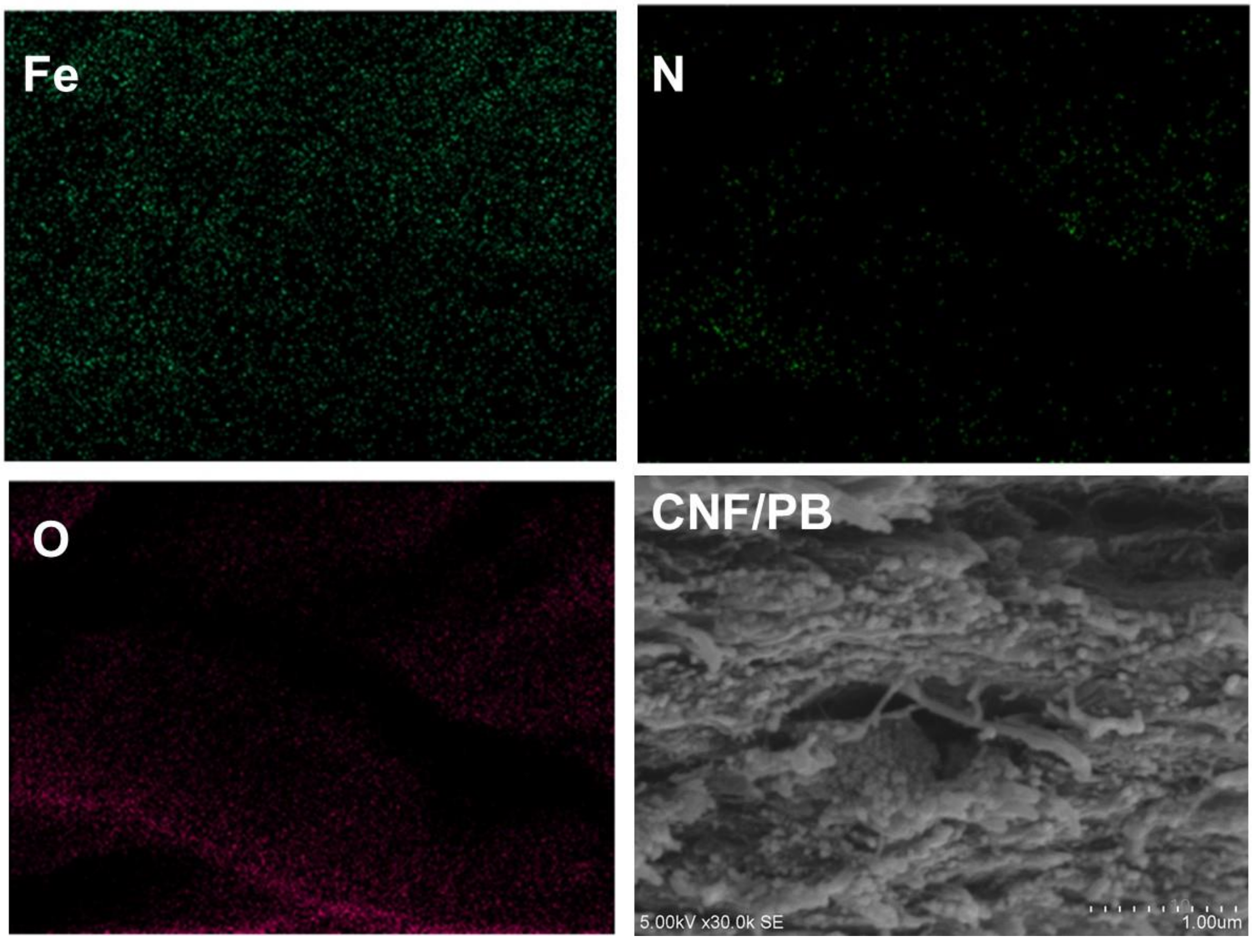

Figure S5: EDS mappings of a CNF/PB complex powder sample.

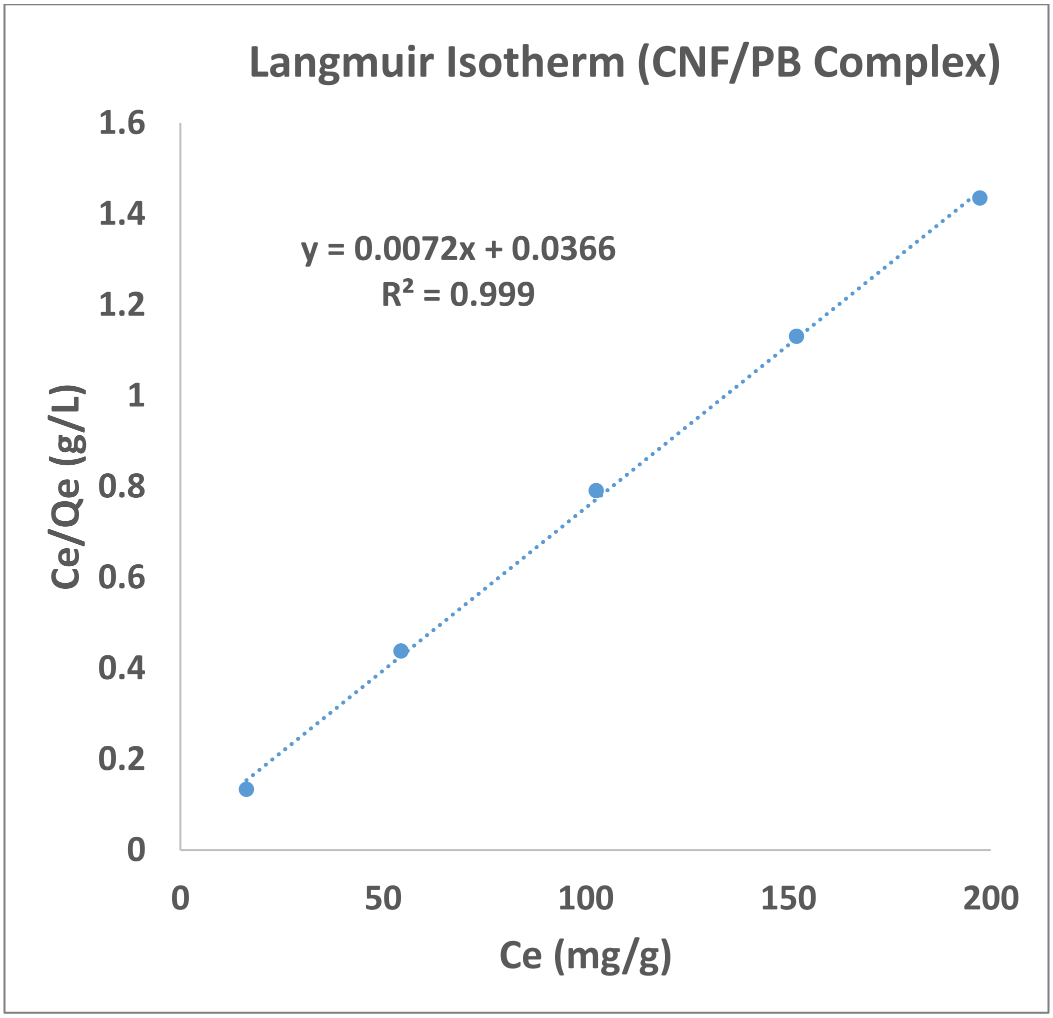

Figure S6. Linear fitting of CNF/PB complex powder Cs adsorption data on Langmuir isotherm.

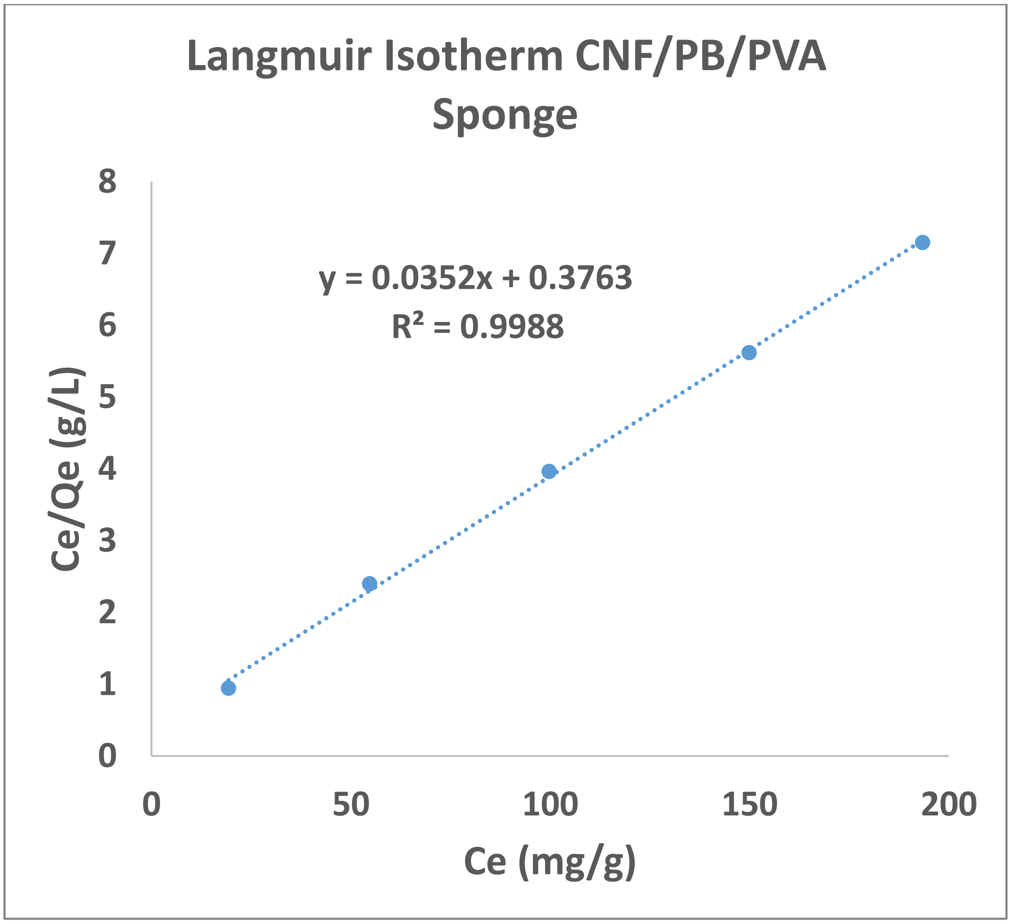

Figure S7. Linear fitting of CNF/PB sponge Cs adsorption data on Langmuir isotherm.

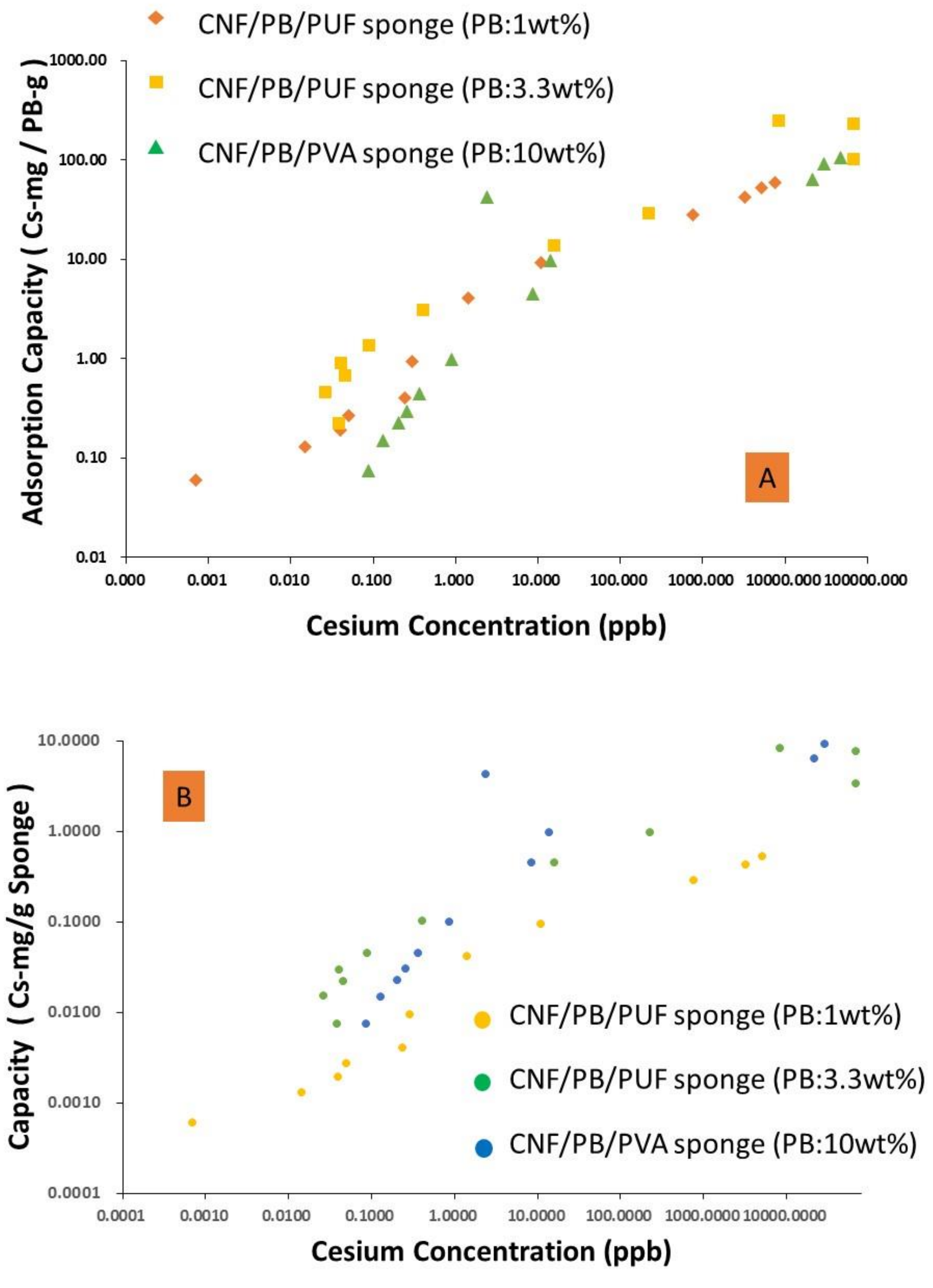

Figure S8. Low level Cesium adsorption experiments using CNF/PB sponges: A) Adsorption capacity based on PB and B) adsorption capacity of Cs based on the different sponges.

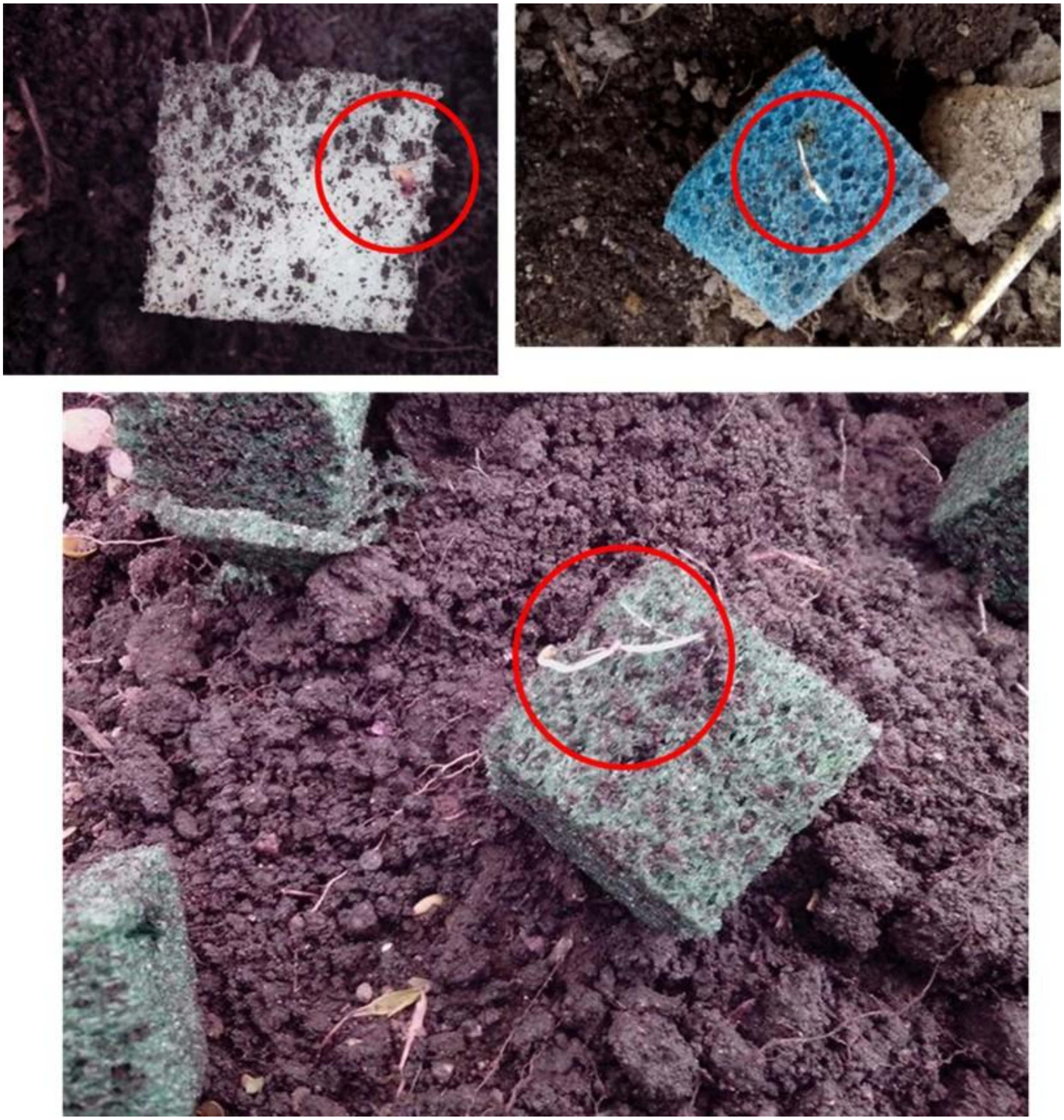

Figure S9. Seedling stage of plants in the sponge after one week.

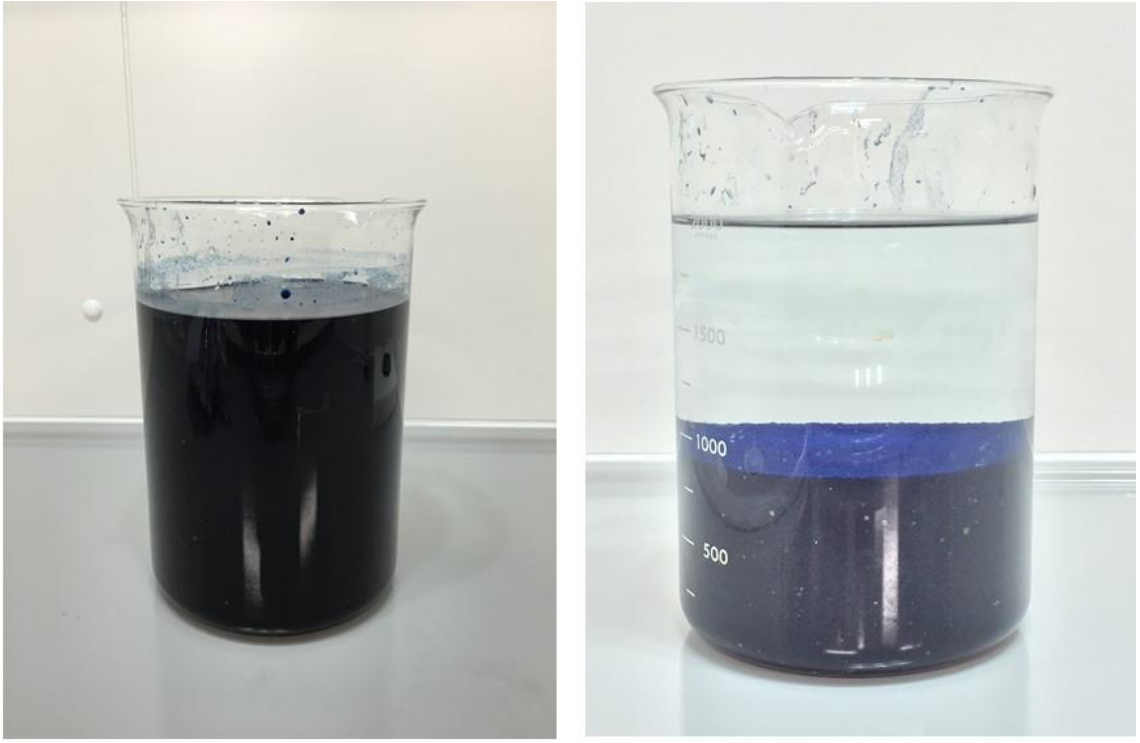

CNF/PB in water (Zero hour) CNF/PB in water (One hour)

Figure S10. CNF/PB complex colloids settling property.

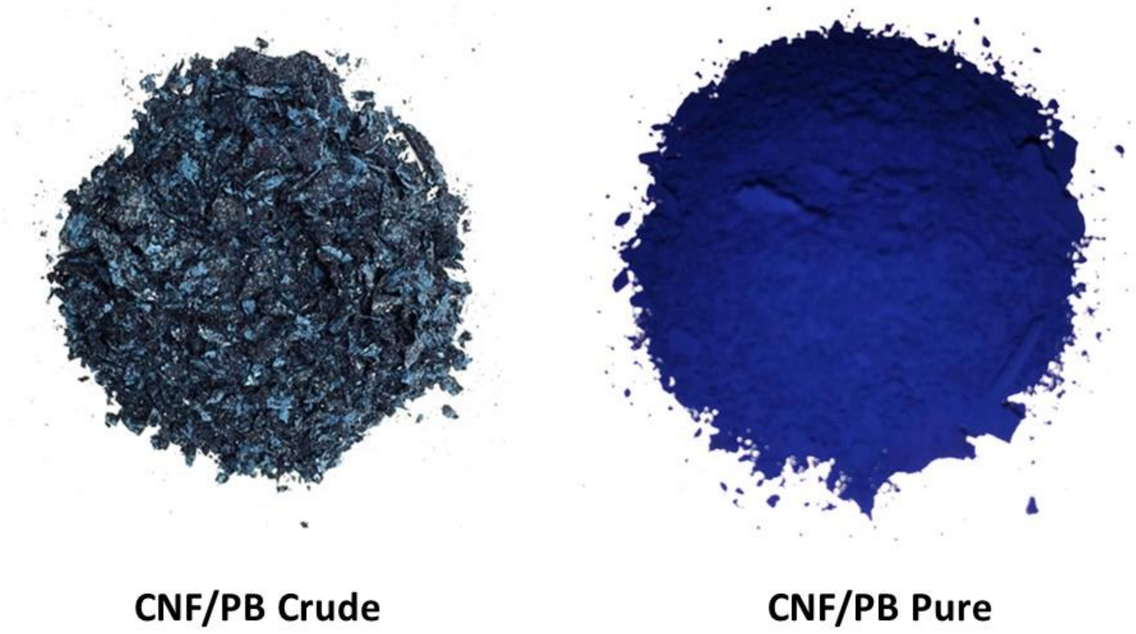

Figure S11. CNF/PB complex powder photo images.

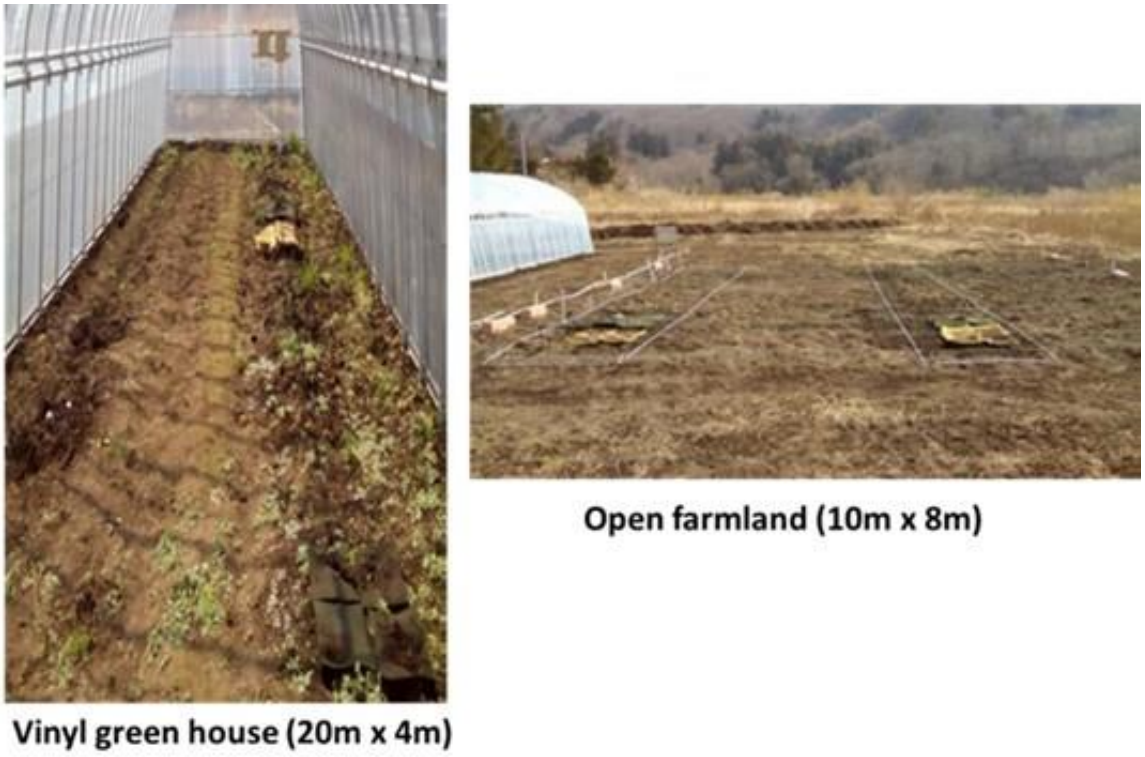

Figure S12. Selected area for practical feasibility studies (At Fukushima, Japan).

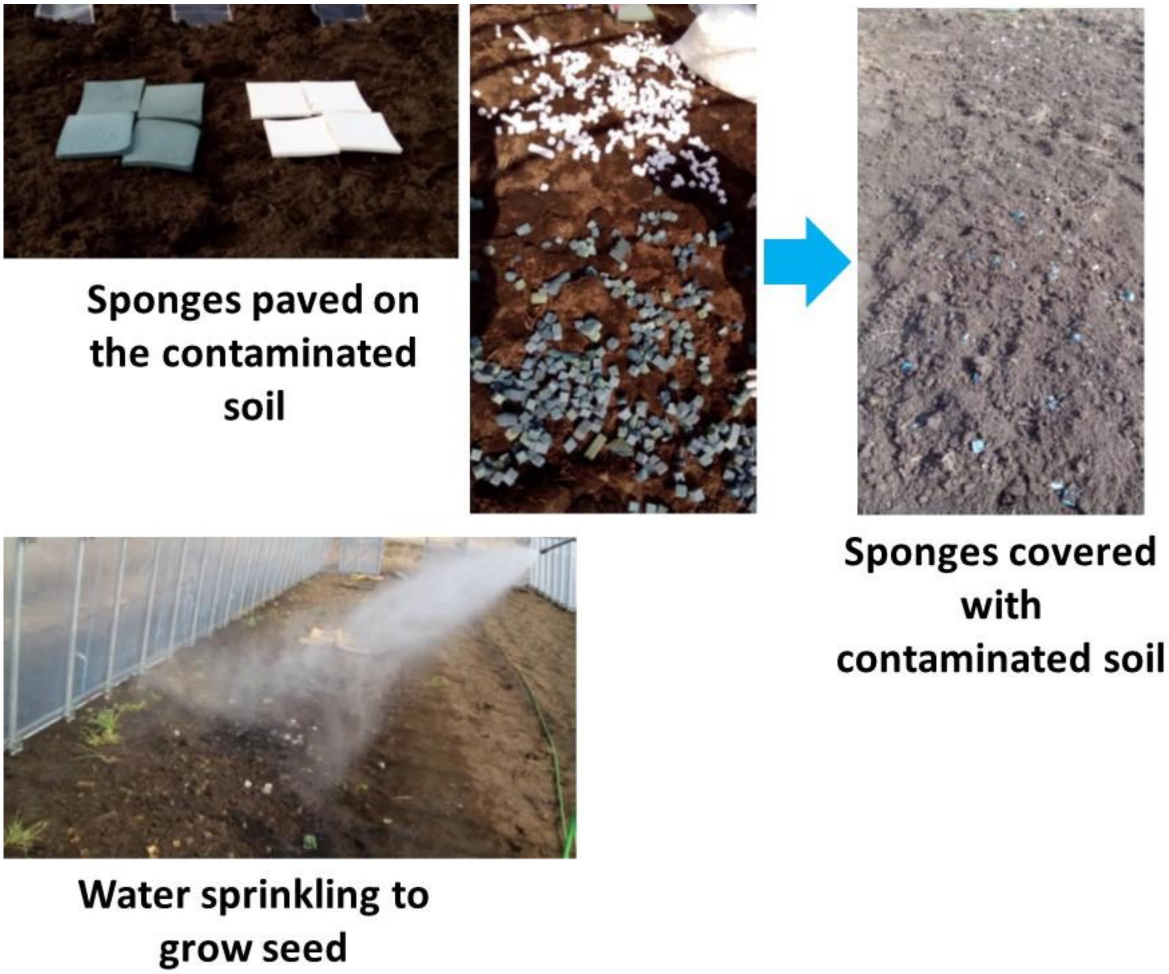

Figure S13. Practical set up of decontamination using the CNF/PB sponge with seeds.
